# Optimism and mental imagery: A possible cognitive marker to promote well-being?

**DOI:** 10.1016/j.psychres.2012.09.047

**Published:** 2013-03-30

**Authors:** Simon E. Blackwell, Nathaly Rius-Ottenheim, Yvonne W.M. Schulte-van Maaren, Ingrid V.E. Carlier, Victor D. Middelkoop, Frans G. Zitman, Philip Spinhoven, Emily A. Holmes, Erik J. Giltay

**Affiliations:** a MRC Cognition and Brain Sciences Unit, Cambridge, UK; b Department of Psychiatry, University of Oxford, Oxford, UK; c Leiden University Medical Centre, Department of Psychiatry, Leiden, The Netherlands; d Institute of Psychology, Leiden University, Leiden, The Netherlands

**Keywords:** Positive imagery, Future thinking, Mental simulation

## Abstract

Optimism is associated with a range of benefits not only for general well-being, but also for mental and physical health. The development of psychological interventions to boost optimism derived from cognitive science would have the potential to provide significant public health benefits, yet cognitive markers of optimism are little understood. The current study aimed to take a first step in this direction by identifying a cognitive marker for optimism that could provide a modifiable target for innovative interventions. In particular we predicted that the ability to generate vivid positive mental imagery of the future would be associated with dispositional optimism. A community sample of 237 participants completed a survey comprising measures of mental imagery and optimism, and socio-demographic information. Vividness of positive future imagery was significantly associated with optimism, even when adjusting for socio-demographic factors and everyday imagery use. The ability to generate vivid mental imagery of positive future events may provide a modifiable cognitive marker of optimism. Boosting positive future imagery could provide a cognitive target for treatment innovations to promote optimism, with implications for mental health and even physical well-being.

## 1 Introduction

Why is it that some people see the future as bright and full of potential, whereas for others it holds only uncertainty or apprehension? Dispositional optimism refers to the tendency to have generalized positive expectancies about the future (Carver et al., 2010). Most people show an “optimism bias”, expecting positive events rather than negative events to happen in the future, even without supporting evidence (Weinstein, 1980).

It has been argued that optimism is adaptive and an important product of human evolution (Sharot, 2011). An increasing body of evidence suggests that optimism has an impact not only on general well-being, but also on mental and physical health (Carver et al., 2010). Longitudinal studies have demonstrated that higher levels of optimism are associated with lower cumulative incidence of depression symptoms over a 15-year period (Giltay et al., 2006b), with reduced risk of future cardiovascular disease in a range of populations (Giltay et al., 2006a; Tindle et al., 2009; Boehm et al., 2011), and even with reduced rate of death (Giltay et al., 2004). Optimism is thus linked to positive outcomes in areas that represent huge public burdens such as depression and cardiovascular disease (World Health Organization, 2008). In the context of the need to develop inexpensive and accessible treatment options (Simon and Ludman, 2009), optimism presents a target for a low-intensity psychological interventions in these high-priority areas.

Although some potential psychological interventions to increase optimism have been described (e.g. Riskind et al., 1996; Meevissen et al., 2011), the development of novel interventions for optimism is most likely to be successful if it is rooted in an understanding of the basic underlying processes, and this is currently lacking. Developing an understanding of the cognitive and emotional processes underlying optimism using an “experimental medicine” approach (Rutter and Plomin, 2009) could drive more targeted treatment innovation. This corresponds to the “basic science discovery” phase in the development of new interventions (Thornicroft et al., 2011).

A potential neural substrate for optimism has been suggested. Sharot et al. (2007) found increased activation in the right anterior cingulate cortex (rACC) when participants imagined positive future events, compared to when they imagined negative future events. Furthermore, this relative level of rACC activation was greater for participants with higher levels of self-reported optimism. While the identification of brain regions per se does not easily lend itself to novel treatment development, this study suggests a potentially modifiable cognitive marker: the paradigm used involved the generation of mental imagery, that is, imagining autobiographical episodes.

We propose that a candidate cognitive marker for optimism is the ability to generate vivid mental images of positive events occurring in the future. Imagining the future may play a key role in our day-to-day functioning and has been the subject of much recent research interest (e.g. Schacter et al., 2008; Addis et al., 2009; Crisp et al., 2011; D'Argembeau et al., 2011). Compared to verbal thought, mental imagery has a powerful effect on emotion (Holmes and Mathews, 2005; Holmes et al., 2008), and thus mental images may be a particularly powerful form of future thinking. What evidence might support our hypothesis? Sharot et al. (2007) found that participants reporting higher levels of optimism were more likely to expect the positive events they imagined to happen closer in the future than negative events, and were more likely to imagine them with a greater sense of “pre-experiencing”. On the other hand, people with depressed mood showed reduced ability to generate vivid mental images of positive future events (Holmes et al., 2008). Further, Morina et al. (2011) found that patients with major depressive disorder and those with anxiety disorders showed reduced ability to generate vivid mental images of positive future events compared to healthy controls, and also rated the events as less likely to occur in the near future.

Support for a link between imagery of the future and optimism also comes from experimental studies that have investigated the potential of imagery tasks to boost optimism. Meevissen et al. (2011) investigated the impact on optimism of practising a “Best Possible Self” (BPS) imagery exercise every day for 2 weeks. This built on work by Fosnaugh et al. (2010) demonstrating that optimism was manipulable in an experimental setting, and a subsequent study by Peters et al. (2010) that showed an immediate impact on optimism of engaging in a BPS imagery exercise. The BPS imagery exercise involved imagining a future self in which everything had turned out in the most optimal way. In the study by Meevissen et al. (2011), participants were asked to repeat the imagery exercise for 5 min each day at home over a 2-week period. In a control condition participants instead carried out the imagery exercise about their daily activities in the past 24 h. Participants in the BPS imagery condition (*n*=28) showed significant increases in self-reported optimism over the 2 weeks, whereas participants in the control condition (*n*=26) did not show this increase. This therefore provides some evidence that engaging in positive future imagery may lead to increases in optimism in the short term, whereas engaging in past imagery does not.

There is therefore convergent evidence from both ends of the optimism spectrum to suggest that positive future imagery may be important, and from experimental studies that deliberate engagement in positive future imagery can increase optimism. However, a fundamental part of the puzzle is missing. That is, is optimism in fact associated with greater ability to generate vivid mental images of positive events in the future? At first glance it may sound self-evident that people who can more easily imagine a positive future would be more optimistic, but strikingly this has not been put to the test, and in fact the widely used measure of optimism, the Life Orientation Test-Revised (LOT-R; Scheier et al., 1994), makes no mention of positive imagination per se. Even in the study by Meevissen et al. (2011) described above, as both the experimental conditions involved engaging in imagery, the study cannot demonstrate whether the imagery component of the exercise was crucial for the effects of the task (as opposed to simply thinking about positive futures), or whether the increase in optimism observed in the experimental group was the result of specific cognitive changes such as increased accessibility of positive future imagery. The key hypothesised link between optimism and vividness of positive future imagery therefore remains untested.

The current study aimed to test the hypothesis that within a community sample, higher levels of optimism would be associated with the ability to generate more vivid mental imagery of positive future events, as measured by vividness ratings on the Prospective Imagery Test (PIT). We predicted that this relationship would remain significant when adjusting for other potentially confounding variables. The study further aimed to extend the findings of Sharot et al. (2007) by investigating the relationship between the sense of likelihood and pre-experiencing of future imagery and optimism, by adding ratings of likelihood and experiencing to the PIT.

## 2 Method

### 2.1 Participants

The study sample was drawn from the Routine Outcome Monitoring (ROM; de Beurs et al., 2011) reference study (Schulte-van Maaren et al., 2012). The ROM reference study comprised a population-based sample of Dutch participants aged 18–65, randomly selected from registration systems of eight general practitioners (GPs) in the province of South-Holland, the Netherlands. In the Netherlands, 99.9% of the general population is registered with a GP, and thus non-consulting GP patients provide a good representation of the general population. The ROM reference group was stratified according to the composition of the ROM patient group (regarding age, gender, and urbanization). As a reference sample, participants with cognitive difficulties such as dementia or who had received treatment for a psychiatric disorder within the past 6 months were excluded. The 547 people in the reference study who had agreed to be contacted for research were invited by letter to participate, with the questionnaires and return envelope enclosed, and 258 elected to take part.^1^ Twenty-one participants returned incomplete questionnaires and were excluded, leaving a final sample of 237 (152 men and 85 women).

### 2.2 Measures

Socio-demographic variables (e.g. age, gender, education) were collected as part of the ROM reference study. For the current study, participants further completed the following questionnaires.

#### 2.2.1 Life Orientation Test-Revised (LOT-R; Scheier et al., 1994).

This 10-item questionnaire was used to assess dispositional optimism. Items were rated on a 5-point scale from 0 (*strongly disagree*) to 4 (*strongly agree*). Three items were positively worded (e.g. “I'm always optimistic about my future”), and three were negatively worded and reverse-scored (e.g. “I hardly ever expect things to go my way”). Four items were filler, and participants' responses to these were not used in calculating their score. Higher total scores (ranging from 0 through 24) were indicative of higher levels of optimism. Although some have argued that the positively worded and negatively worded items on the LOT-R should be scored separately to generate separate optimism and pessimism scales (e.g. Kubzansky et al., 2004), we used the original scoring as described by the authors of the scale, consistent with other studies investigating optimism in the context of mental imagery (e.g. Sharot et al., 2007; Meevissen et al., 2011). The LOT-R has been used in numerous studies investigating optimism (Carver et al., 2010), and Scheier et al. (1994) report acceptable internal consistency (*α*=0.78), as well as good convergent and discriminant validity. Internal consistency in our sample was acceptable (*α*=0.77).

#### 2.2.2 Prospective Imagery Test (PIT; Stöber, 2000; Holmes et al., 2008).

The PIT is a measure of deliberately generated positive and negative mental images of potential future events. Participants were presented with 10 positive and 10 negative future scenarios and generated a mental image of each. Participants rated the vividness of each image on a scale from 1 (*no image at all*) to 5 (*very vivid*). To obtain further information about the quality of imagery generated (cf. Sharot et al., 2007), participants rated their perceived “likelihood” of each event occurring in the near future from 1 (*not at all*) to 7 (*completely*), and to what extent they felt that they were “experiencing” each event while imagining it from 1 (*not at all*) to 7 (*completely*). In the current study we were primarily interested in responses to the positive items, and included the negative items to control for general ability to generate future imagery. As internal consistency had not previously been reported for subscales of the PIT, we calculated Cronbach's *α* for our sample. All subscales demonstrated good internal consistency (0.83<*α*<0.90).

#### 2.2.3 Spontaneous Use of Imagery Scale (SUIS; Reisberg et al., 2003).

This 12-item questionnaire was included to control for everyday imagery use. Participants rated items such as “When I think about visiting a relative, I almost always have a clear mental picture of him or her” on a scale from 1 (*never appropriate*) to 5 (*always appropriate*). Reisberg et al. (2003) report excellent internal consistency (*α*=0.98) and good convergent validity. Internal consistency in our sample was good (*α*=0.81).

## 3 Results

Table 1 presents descriptive statistics for socio-demographic information^2^ and their zero-order correlations with scores on the LOT-R.

### 3.1 Optimism and vividness of positive future imagery

Hierarchical regression was used to test our key hypothesis that ability to generate vivid images of *positive* future scenarios would predict optimism when controlling for other variables. In step 1, socio-demographic variables were entered. In step 2, score on the SUIS and vividness for negative items on the PIT were entered, to control for the everyday use of imagery and general ability to generate vivid mental images. In step 3, vividness ratings for the *positive* items of the PIT were entered. Table 1 summarises the regression. Adding the SUIS and vividness for negative items on the PIT in step 2 did not significantly improve the fit of the model (Δ*R*^2^=0.003, Δ*F*(2, 224)<1). However, adding the vividness ratings for the positive items of the PIT in step 3 significantly improved the fit of the model (Δ*R*^2^=0.19, Δ*F*(1, 223)=63.81, *P*<0.001). In the final model, higher ratings for vividness of positive items on the PIT were significantly associated with higher scores on the LOT-R, and higher ratings for vividness of negative items on the PIT were significantly associated with lower scores on the LOT-R. The only other variable with a significant regression coefficient was age. Examination of residuals plots revealed no multivariate outliers, and no problems with collinearity were identified (inspection of Tolerance/Variance Inflation Factors; Clark-Carter, 2010). In summary, the regression supported our key hypothesis by demonstrating that higher vividness for images generated of positive future events (PIT) was associated with higher levels of dispositional optimism (LOT-R) even when controlling sociodemographic factors, everyday use of imagery and vividness of negative future imagery.

### 3.2 Optimism and other qualities of future imagery

Table 2 presents descriptive statistics for the measures of imagery (SUIS and subscales of the PIT), their inter-correlations, and their zero-order correlations with the LOT-R. Vividness, likelihood and experiencing ratings for positive items of the PIT each correlated significantly with score on the LOT-R, suggesting that each of these qualities of the positive future imagery generated was significantly associated with higher levels of optimism. Conversely, only likelihood and experiencing ratings for negative items of the PIT correlated significantly with score on the LOT-R, while vividness ratings for negative items on the PIT and score on the SUIS did not correlate significantly with score on the LOT-R.

## 4 Discussion

This study is the first, to our knowledge, to test the prediction that the ability to generate vivid positive mental imagery of the future is associated with dispositional optimism. Data from a community sample supported this association. The relationship was significant when controlling for socio-demographic factors, general use of imagery, and vividness of negative future imagery. This suggests that further research is warranted in investigating positive future imagery as a potential cognitive marker for optimism and a target for treatment innovation or even prevention in, for example, depression and cardiovascular disease (Giltay et al., 2004, 2006b).

In addition to imagery vividness, we also considered the characteristics of likelihood and pre-experiencing. The positive future images of more optimistic participants were not only more vivid, but also associated with a sense of greater likelihood of occurring in the near future, and of “pre-experiencing” the imagined event (i.e. the sense of it happening now in the present). This extends the findings of Sharot et al. (2007), who found in their functional magnetic resonance imaging (fMRI) study that more optimistic participants rated imagined positive events as more likely to happen closer in the future than negative events, and experienced these positive events with a greater sense of pre-experiencing. We were able to investigate such relationships within a larger representative sample, and controlling for individual differences in general imagery use.

Additionally, without the constraint of a subtraction condition, as in an fMRI study, we could examine the separate relationships between optimism and the characteristics of positive and negative future imagery on the PIT. We found a robust relationship between vividness of positive imagery and optimism, indicating that the more vividly someone could imagine a future achievement, for example, the more optimistic the individual was. A significant association between vividness of negative imagery and optimism only emerged once the relationship between positive imagery vividness and optimism was controlled for in the regression. For likelihood and experiencing ratings, both positive and negative imagery were related to optimism. That is, optimists showed a greater tendency to endorse, for example, the likelihood of imagined future positive relationships, yet rated imagined future disputes with friends as less likely. Optimists were also more likely to have a greater sense of “pre-experiencing” when imagining a possible positive future event, but a weaker sense of pre-experiencing if the imagined future event was negative.

The link between *positive* future imagery and optimism may be understood within a cognitive science framework and with reference to the hypothesised role of mental simulation in thinking about and planning the future (Schacter et al., 2008), in conjunction with the role of emotional valence. That is, when someone thinks about events in the future, it is likely to be in the form of mental imagery. As mental imagery has a particularly strong link to emotion (Holmes and Mathews, 2005), cognitions about the future that take an imagery form may have a particularly strong impact on the affective tone of the perceived future. The relative accessibility and clarity of positive vs. negative mental images will impact differentially on the expectations an individual has for the future (Sharot et al., 2007). *Positive* future images may therefore lead to increased optimism.

However, a greater tendency to use imagery generally in everyday life (irrespective of emotional valence) would not be expected to be associated with higher levels of optimism as this would apply equally to both positive and negative future thinking. This accords with the lack of relationship between the SUIS and LOT-R in our study, and also with the findings of Meevissen et al. (2011), that merely practising imagery alone (their daily activities condition) did not lead to increases in optimism. Relatedly, it has been argued that therapeutic approaches need to focus on boosting positive aspects of experience and not simply reducing negative aspects (cf. MacLeod and Moore, 2000; MacLeod, 2012). Consistent with this, our results suggest that although negative future imagery may be a valid target for interventions to reduce negative affect, enhancing the ability to vividly imagine positive future events is likely to be more fruitful in increasing positive features such as optimism. That is, if someone is currently unable to imagine a positive future, then simply reducing the vividness of their negative mental images of the future is likely to be of limited impact, and rather it is their positive future imagery that needs boosting.

Limitations of the study include its correlational nature, which means that issues of causation cannot be explored. Further, fewer than half of those invited to take part in the study elected to take part, and so there could be a self-selecting bias in the study sample; for example, individuals with less awareness of mental imagery may have declined to participate. Additionally, the characteristics of those who declined to form part of the ROM reference sample from which this study sample was drawn have not been analysed, and so although it was designed to be representative, the design's success cannot be assumed. It will therefore be useful to replicate the main findings of this study in other community samples. Future studies investigating the relationship between future imagery and optimism could also benefit from including a measure of depression, in order to investigate whether the relationship found in the current study is independent of current mood. Although we measured the ability of participants to generate vivid future imagery, we did not measure how much they spontaneously generated or experienced such imagery in their daily life. We might expect optimists to not only be better at generating vivid images of positive future events, but to also experience more of such positive future imagery on a moment to moment basis. This would be useful to investigate in future studies, and would also suggest that interventions to boost optimism should aim to increase not only the ability to generate vivid positive future images, but also the likelihood of automatically generating such images in the context of ambiguous cues in their daily lives (cf. Pictet et al., 2011).

This study represents a critical first step in identifying a modifiable cognitive marker underlying optimism. This cognitive approach complements research exploring neural (Sharot et al., 2007, 2011) or genetic (Fox et al., 2009; Fox, 2012) associates of optimistic thinking styles. The focus on positive imagery fits within a broader literature exploring its potential relevance across a range of areas, such as understanding memory in depression (Werner-Seidler and Moulds, 2011) or its use in emotion regulation (Jacob et al., 2011). It is important to remember that reduced optimism may be a reflection of societal disadvantage, and in this context a cognitive intervention should not be seen as an alternative to tackling broader societal problems. However, there are a range of conditions in which a novel intervention for optimism may be extremely valuable. Current findings suggest that innovative imagery-based interventions to increase optimism should focus on boosting the ability to vividly imagine positive events in the future, e.g. via a computerized, potentially even internet-delivered, intervention (Blackwell and Holmes, 2010).

In summary, why is it that some people see the future as bright and full of potential? Current results suggest that when optimists imagine the future, they can literally see, in their mind's eye, vivid scenes of positive possibilities. We hope that this research suggests future avenues for research to develop novel interventions that will enable more people to take such an optimistic outlook, and thus improve mental health and physical well-being.

## Figures and Tables

**Table 1:** Descriptive statistics and hierarchical multiple regression analyses predicting scores on the Life Orientation Test-Revised via socio-demographic data and measures of imagery.

Predictor	*M* (S.D.)	*r*_0_	Model 1	Model 2	Model 3
			*β*	*β*	*β*
Age (years)	43.11 (12.61)	0.05	0.09	0.09	0.15⁎
Gender	*n* (%)				
Female	85 (36%)	0.02	−0.02	−0.01	−0.06
Married/cohabiting	164 (69%)	0.22⁎⁎⁎	0.07	0.07	0.08
Living alone	42 (18%)	−0.27⁎⁎⁎	−0.19⁎	−0.19⁎	−0.13
University level education	196 (83%)	0.08	0.10	0.10	0.05
Dutch nationality	231 (98%)	0.07	0.08	0.09	0.03
Current smoker	44 (19%)	−0.09	−0.05	−0.05	−0.003
No or hardly any alcohol use	23 (10%)	−0.10	−0.05	−0.04	−0.034
Health status					
Self-rated “healthy”	221 (93%)	0.20⁎⁎	0.16⁎	0.15⁎	0.03
Serious illness diagnosed	47 (20%)	0.07	0.06	0.06	0.04
SUIS	35.15 (8.56)	−0.03		0.01	−0.09
PIT-Negative					
Vividness	2.81 (0.97)	−0.08		−0.06	−0.23⁎⁎⁎
PIT-Positive					
Vividness	3.88 (0.68)	0.44⁎⁎⁎			0.52⁎⁎⁎
Adjusted *R*^2^			0.09	0.08	0.28
Δ*R*^2^				0.003	0.19
*F* for Δ*R*^2^				0.41	63.81⁎⁎⁎
Model *F*			3.26⁎⁎	2.77⁎⁎	8.18⁎⁎⁎

*N*=237. Model 1 includes socio-demographic variables only. Model 2 additionally includes control imagery variables. Model 3 additionally includes positive future imagery vividness. *r*_0_=zero order correlations. SUIS=Spontaneous Use of Imagery Scale. PIT-Negative/Positive=Prospective Imagery Test Negative/Positive items.

⁎ *P*<0.05.

⁎⁎ *P*<0.01.

⁎⁎⁎ *P*<0.001.

**Table 2:** Descriptive statistics, and Pearson product-moment correlations between scores on measures of optimism, everyday use of imagery, and subscales of the Prospective Imagery Test.

	*M* (S.D.)	1	2	3	4	5	6	7
1. LOT-R	17.08 (4.35)							
2. SUIS	35.15 (8.56)	−0.03						

PIT-Positive								
3. Vividness	3.88 (0.68)	0.44⁎⁎⁎	0.22⁎⁎					
4. Likelihood	4.72 (0.99)	0.45⁎⁎⁎	0.12	0.75⁎⁎⁎				
5. Experiencing	4.54 (1.18)	0.30⁎⁎⁎	0.21⁎⁎	0.69⁎⁎⁎	0.78⁎⁎⁎			

PIT-Negative								
6. Vividness	2.81 (0.97)	−0.08	0.18⁎⁎	0.34⁎⁎⁎	0.05	0.10		
7. Likelihood	2.86 (0.97)	−0.24⁎⁎⁎	0.11	−0.001	0.007	0.04	0.62⁎⁎⁎	
8. Experiencing	2.84 (1.25)	−0.15⁎	0.15⁎	0.17⁎	0.09	0.33⁎	0.70⁎⁎⁎	0.72⁎⁎⁎

*N*=237. LOT-R=Life Orientation Test-Revised. SUIS=Spontaneous Use of Imagery Scale. PIT-Negative/Positive=Prospective Imagery Test Negative/Positive items.

⁎ *P*<0.05.

⁎⁎ *P*<0.01.

⁎⁎⁎ *P*<0.001.

## Footnotes

1 There were no significant differences in socio-demographic characteristics of those who did or did not respond to the invitation to take part in the study, with the exception of nationality and age. Thus, the sample for the current sample was broadly representative of the ROM reference sample as a whole, except that responders were significantly more likely to be Dutch, *χ*^2^(1,457)=6.52, *P*=0.01, and were significantly older, *t*(545)=4.90, *P*<0.001, than non-responders. All other *P*s were >0.10, with the exception of gender, where there was a trend for a greater proportion of responders to be male than non-responders, *χ*^2^(1,457)=3.54, *P*=0.06. The ROM reference study had also included the LOT-R, and there were no differences between responders and non-responders on this prior administration, *t*(544)<1.

2 For the regression analysis, socio-demographic information was coded into binary variables: male vs. female; married/cohabiting vs. unmarried; living alone vs. living with others; university level education vs. lower than university level education; Dutch nationality vs. other nationality; current smoker vs. current non-smoker; no or hardly any alcohol consumption vs. at least a glass of alcohol per week; self-rated health as “healthy” vs. self-rated health as “unhealthy”; lifetime diagnosis from a doctor of serious illness (cardiovascular disease, stroke, diabetes, cancer, asthma, emphysema, chronic bronchitis) vs. never received such diagnoses. We included variables such as marital status and self-rated health that have previously shown associations with optimism (Giltay et al., 2007) in order to examine whether a strong relationship between our cognitive variable of interest (vividness of positive future imagery) and optimism remained even when controlling for these other associations.
